# Tumeur fibreuse solitaire pleurale

**DOI:** 10.11604/pamj.2015.22.339.7357

**Published:** 2015-12-08

**Authors:** Khaoula Elatiqi, Najiba Yassine

**Affiliations:** 1 Service des Maladies Respiratoires, CHU Ibn Rochd, Casablanca, Maroc

**Keywords:** tumeur fibreuse solitaire, plèvre, chirurgie, solitary fibrous tumor, pleura, surgery

## Image en médecine

Un patient âgé de 48 ans, tabagique et cannabique chronique, a consulté pour une toux sèche associée à une douleur thoracique sous mammaire gauche qui persistait depuis 3 mois, dans un contexte de conservation de l’état général. L'examen à l'admission retrouvait une matité du tiers supérieur de l'hémithorax gauche. Le scanner thoracique avec injection a objectivé une grosse masse pulmonaire apico-dorsale gauche arrondie bien limitée, mesurant 94x77x83 millimètres, renfermant des calcifications fortement rehaussées après injection de produit de contraste et arrivant au contact de l'aorte descendante avec un liseré graisseux de séparation. La bronchoscopie a montré un état inflammatoire du premier degré diffus, sans tumeur visible. Le bilan d'extension était négatif. Le patient a été opéré par thoracotomie postéro-latérale droite. L'exploration chirurgicale a objectivé une masse tumorale au dépend de la plèvre pariétale de 15 cm de diamètre, ovalaire, prenant tout l'apex, adhérente au lobe supérieur droit et au lobe inférieur et laissant le parenchyme pulmonaire intact. Une résection complète de la masse a été réalisée avec bonne évolution en postopératoire. L'examen anatomopathologique de la pièce opératoire a conclu à une tumeur fibreuse solitaire avec une forte expression de l'anticorps anti-CD34. L’évolution a été bonne après un recul de deux mois.

**Figure 1:**
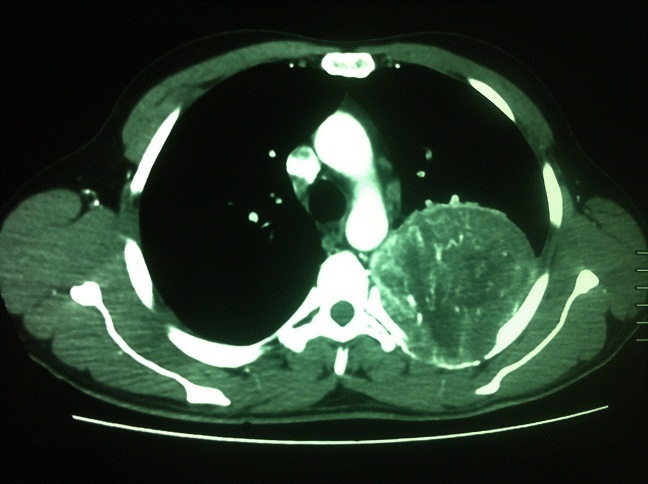
Scanner thoracique montrant une masse apico-dorsale gauche

